# Glutathione S-Transferase M1 and T1 Gene Deletions and Susceptibility to Acute Lymphoblastic Leukemia (ALL) in adults

**DOI:** 10.12669/pjms.343.14911

**Published:** 2018

**Authors:** Alveena Zehra, Sitwat Zehra, Muhammad Ismail, Abid Azhar

**Affiliations:** 1 Alveena Zehra, PhD Student (MSc). The Karachi Institute of Biotechnology and Genetic Engineering (KIBGE), University of Karachi, Karachi, Pakistan; 2 Sitwat Zehra, PhD. The Karachi Institute of Biotechnology and Genetic Engineering (KIBGE), University of Karachi, Karachi, Pakistan; 3 Muhammad Ismail, PhD. Institute of Biomedical and Genetic Engineering (IBGE), Islamabad, Pakistan; 4 Abid Azhar, DSc, PhD. The Karachi Institute of Biotechnology and Genetic Engineering (KIBGE), University of Karachi, Karachi, Pakistan

**Keywords:** Acute Lymphoblastic Leukemia, ALL, *GSTM1*, *GSTT1*, Genetic, Polymorphism

## Abstract

**Objective:**

Biotransformation of xenobiotics are critical for their metabolism and removal from the body which is carried out by xenobiotic metabolizing enzymes. Individuals carrying variants of genes that encode these enzymes have an altered ability to metabolize xenobiotics which may lead to an increased risk of acute lymphoblastic leukemia. The current study aimed to investigate the impact of *GSTM1* and *GSTT1* gene deletions in causing predisposition to adult ALL.

**Methods:**

The current case-control study involved 62 adult ALL patients and 62 age and gender matched healthy controls. Whole blood samples processed with standard phenol chloroform protocol for DNA isolation were genotyped using multiplex PCR approach for simultaneous identification of *GSTM1* and *GSTT1* deletions. The genotype frequency obtained for patients was compared to controls using odds ratio and chi-square.

**Results:**

The null genotype frequency of *GSTM1* and *GSTT1* in a group of adult ALL patients from Pakistan were 47% and 11% respectively. Deletion of *GSTM1* and *GSTT1* did not show statistically significant association with adult ALL (*p*=0.86 and *p*=0.35 respectively). The combined *GSTM1/GSTT1* deletion was observed in 2% patients and was not significantly associated with ALL in adults (*p*=0.85).

**Conclusions:**

The results reveal that homozygous null polymorphism of *GSTM1* and *GSTT1*genes does not influence ALL susceptibility among adult patients. Cancer susceptibility associated with *GST* polymorphism varies with ethnic and geographic differences. Therefore, further investigation on different populations is needed to understand the role of these genetic variations in modifying adult ALL risk.

## INTRODUCTION

Acute lymphoblastic leukemia (ALL) is a blood malignancy distinguished by an excessive buildup of lymphoid progenitor cells in blood and bone marrow. ALL constitutes one fourth of all cancer cases occurring in childhood making it the most common pediatric cancer.^1^ ALL among adults accounts for less than 1% of total cancer cases.^2^ According to American Cancer Society, 5960 new cases including both children and adults have been estimated to be diagnosed with ALL with over 1470 deaths within the United States in 2018.^3^ While exceptional progress has been made in pediatric ALL outcomes over last few decades with long term survival rates exceeding 80% in recent reports, outcomes for adult ALL remain considerably poor.^4^,^5^

The development of ALL includes both genetic and environmental factors with DNA damage in hematopoietic precursor cells being acrucial step.^6^ Reactive oxygen species (ROS) generated by environmental toxins and chemical carcinogens result in DNA damage.^7^ Being substrates of carcinogen metabolizing enzymes, the xenobiotics influence their carcinogenic effect depending on a person’s ability to activate or inactivate them by conjugation and detoxification of these compounds.^8^ Variation in genes that encode carcinogen metabolizing enzymes may therefore explain the differences between the individual’s capacity to metabolize different chemical carcinogens and have thus received a considerable level of attention with respect to cancer development.

The glutathione S-transferases (GSTs) constitute an enzyme super family responsible for detoxification of carcinogens. Detoxification represents the second phase of carcinogen metabolism in human body, followed by bioactivation of procarcinogens (phase I). GSTs detoxify reactive intermediates produced during the first phase of metabolism by conjugating soluble glutathione with them.^9^ Furthermore, GSTs are also involved in protection of DNA against the ROS.^10^ Two members of these, *GSTM1* and *GSTT1*, exhibit null polymorphism indicating homozygous deletion of the genes.^11^ This results in absence of the enzymatic activity. These null genetic variants of GSTs have been reportedto modulate susceptibility tov arious cancers. The role of *GST* deletion has also been studied in the development of hematologic malignancies.^12^,^13^ However, few data has been reported so far for ALL in adults. Therefore, in the present study, the distribution of null genetic variantsof *GSTM1* and *GSTT1*among adult ALL patients in comparison to controls was studiedand their association with the occurrence of adult ALL was examined.

## METHODS

The current case control study was assessed and ethically approved by the Institutional Ethical Committee of the Karachi Institute of Biotechnology and Genetic Engineering (KIBGE), University of Karachi. The study comprised 62 adult ALL cases and an equal number of controls. Patients were recruited from department of oncology, Jinnah Postgraduate Medical Centre (JPMC) Karachi, Pakistan after clinical diagnosis. Questionnaire was filled to collect personal information of patients while medical records were checked to extract their clinical history. Patients with history of other cancers were excluded. Healthy individuals from the population qualified as controls and were matched to cases on age and gender. All participants were provided with an information sheet explaining study objective along with potential risks and benefits associated with their participation prior to their enrolment in this study. A written informed consent declaring their voluntary participation was obtained from them.

### Sample Collection

Five ml of whole blood was withdrawn from patients as well as controls in sterile acid citrate dextrose (ACD) vacutainer for genotype analysis. Collected blood samples were stored at -20°C till further use. They were treated with standard phenol chloroform procedure for genomic DNA isolation.^14^ DNA quality was evaluated by gel electrophoresis and the concentration and purity was analyzed by spectrophotometer respectively.

### Genotype Analysis

Multiplex PCR assay was used to screen out homozygous deletions in *GSTM1* and *GSTT1*genes.^15^ The sense and antisense primers used in the reaction were 5′ GAACTCCCTGAAAAGCTAAAGC 3′ and 5′ GTTGGGCTCAAATATACGGTGG 3′ for *GSTM1*, 5′ TTCCTTACTGGTCCTCACATCTC 3′ and 5′ TCACCGGATCATGGCCAGCA 3′ for *GSTT1*which resulted in 219bp and 459bp fragments respectively. *β-globin* gene was indicative of successful PCR. Primers used for its amplification were 5′ CAACTTCATCCACGTTCACC 3′ and 5′ GAAGAGCCAAGGACAGGTAC 3′ that gave a 268bpamplified product. Multiplex PCR was carried out with 200ng extracted DNA in total reaction volume of 20 µl. The other PCR componentsincluded 1x (NH_4_)_2_SO_4_ buffer, 1.5mM MgCl_2_, 200 μM dNTPs mix, 1.5 units of Taq DNA polymerase, 0.6 μM of sense and antisense primer for *GSTM1*, 0.3 μM of each primer for *GSTT1* and 0.4 μM of each primer for *β globin* gene.PCR cycle conditions included an initial melting step at 94°C for four minutes, 35 PCR cycles, each including 30 s denaturation at 94°C, 35 s annealing at 58°C and 30 s elongation at 72°C, followed by a seven minutes long final elongation step at 72°C. Eight microliters ofthe PCRproducts were loaded on 2.5% agarose gel and electrophoresis was carried outfor 40 min at 100 V. The ethidium bromide stained gel containing amplified fragments wasvisualized under gel documentation system.

### Statistical Analysis

Odds ratio (OR) with 95% confidence interval (CI) was calculated to measure the risk conferred by null allele of *GSTM1* and *GSTT1*for the disease. Chi-square test was performed to test association of homozygous deletion in *GSTM1* and *GSTT1* genes with development of adult ALL using VassarStats, a freely available online tool, under the null hypothesis of “no association”.^16^

## RESULTS

The present study involved 62 adult ALL patients ranging from 15 to 55 years of age. The mean age (±SD) of patients at diagnosis was24.7 ± 1.1 years. Patients under 25 years of age constituted 53% of the total number of cases while 89% of patients were less than 35 years. Patients included both genders but male cases predominated female cases with a ratio of 2.4:1.

### Multiplex PCR Analysis

**Fig.1:**
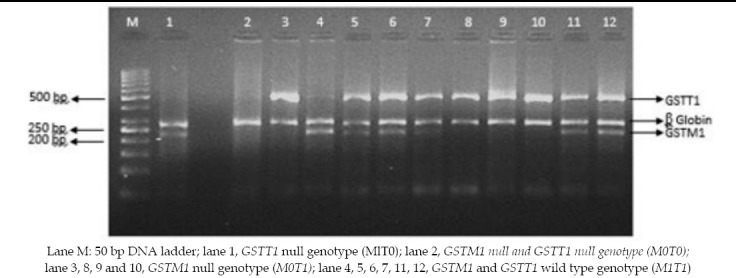
Agarose gel electrophoresis of PCR products for the detection of null polymorphism of *GSTM1* and *GSTT1*

Multiplex PCR assay for co-identification of *GSTM1* and *GSTT1* null polymorphism resulted in banding pattern shown in Fig.1. Absence of a band indicated absence of the respective gene. *GSTM1*deletion resulted in the absence of 219bp amplified product while absence of 459bp fragment meant homozygous deletion of*GSTT1*gene. Amplification of *β-globin* gene resulting in 268bp fragment confirmed the success of PCR when both *GST* genes were deleted.

Lane M: 50 bp DNA ladder; lane 1, *GSTT1* null genotype (MlT0); lane 2, *GSTM1 null and GSTT1 null genotype (M0T0);* lane 3, 8, 9 and 10, *GSTM1* null genotype (*M0T1)*; lane 4, 5, 6, 7, 11, 12, *GSTM1* and *GSTT1* wild type genotype (*M1T1*)

### Genotype Distribution

Frequency distribution of homozygous null polymorphism in *GSTM1* and *GSTT1*genes among patients and controls and the odds of their association with ALL are shown in Table-I. The overall frequency of *GSTM1* and *GSTT1* deletion among study participants (n=124) was46% and 8.7% respectively. *GSTM1* was deleted in 47% adult ALL patients (n=62) and in 45% controls (n=62). The null genotype of *GSTT1* was 11% for cases and 6% for healthy individuals. The Pearson chi-square test revealed neither *GSTM1* deletion nor *GSTT1* null genotype was significantly associated with adult ALL (χ^2^=0.03, *p*=0.86 and χ^2^=0.9, *p*=0.35 respectively). *GSTM1* and *GSTT1* were simultaneously deleted in 1.6% cases as compared to 3.2% controls (Table-II). This difference in genotype frequencies, however, could not attain statistical significance (χ^2^=0.03, *p*=0.85).

**Table-I:** Frequency distribution and odds ratio (95% CI) of GSTM1 and GSTT1 deletion among ALL patients and controls.

Genes	Genotype	Cases (%)	Controls (%)	OR	95% CI
Total (n=62) *GSTM1*	Present	33 (53.2)	34 (54.8)	Ref	
	Null	29 (46.8)	28 (45.2)	1.0671	0.5265 to 2.1628
*GSTT1*	Present	55 (88.7)	58 (93.6)	Ref	
	Null	7 (11.3)	4 (6.4)	1.8455	0.5117 to 6.6553

**Table-II:** Frequency distribution and odds ratio (95% CI) of GSTM1/GSTT1 double deletion among ALL patients and controls

Genes	Genotype	Cases (%)	Controls (%)	OR	95% CI
Total (n=62) *GSTM1/GSTT1*	Present/Present (M1T1)	27 (43.5)	32 (51.6)	Ref	
	Present/Null (M1T0)	6 (9.6)	2 (3.2)	3.5556	0.6625 to 19.0832
	Null/Present (M0T1)	28 (45.2)	26 (41.9)	1.2764	0.6091 to 2.6746
	Null/Null (M0T0)	1 (1.6)	2 (3.2)	0.5926	0.0509 to 6.8984

## DISCUSSION

Glutathione S-transferases take part in detoxification of different carcinogens, environmental toxins and chemotherapeutic drugs. The null genotypes of *GSTM1* and *GSTT1* resulting from homozygous deletion of the respective genes lead to the lack of active enzymes. This loss of enzyme activity affects metabolism of several carcinogens and may therefore influence an individual’s risk of cancer development.*GST* genetic variants are also good candidates for association studies in leukemia as they have a potential to alter metabolism of leukemogens and to cause lack of protection against ROS leading to cellular DNA damage.^17^

The current study evaluating the impact of *GSTM1* and *GSTT1*deletions as predisposing factor of adult ALL revealed that the genotype frequencies of both genes were not significantly higher in ALL patients as compared to healthy controls (47% vs. 45% for *GSTM1* and 11% vs. 6% for *GSTT1*). The use of healthy controls over hospital based controls was intentionally preferred to reduce possible bias in results due to the risk conferred by deletion polymorphism of *GSTM1* and *GSTT1*to non-cancer diseases.

Distribution of *GST* allelesis not uniform across human population and ethnic as well as geographic variations have been observed. In Asians, the frequency of *GSTM1*deletionhas been reported to vary between 42% and 54% (n=1511) while 35% to 52% for *GSTT1* null polymorphism (n=575).^18^ However, these genotype frequencies may not be a true representation of Asian population as the study included data from only 3 countries: Japan, Korea and Singapore. Studies from neighboring countries have reported frequency of *GSTM1* and *GSTT1* deletion to be 45% and 21% in Iranian (n=229) while 33% and 18% in North Indian (n=198) population respectively.^19^,^20^

The current study reports no association of either *GSTM1* null polymorphism (OR, 1.07; 95% CI, 0.53-2.16) or *GSTT1* gene deletion (OR, 1.85; 95% CI, 0.51-6.66) with ALL among adults. Most genetic association studies examining role of *GSTM1* and *GSTT1* deletion as a modulator of ALL risk have focused children affected with ALL. Only a limited number of studies have evaluated this risk with adult ALL owing to the fact that ALL has a higher occurrence in children as compared to adults. A study evaluating 71 adult ALL patients for *GSTM1* and *GSTT1* reported significant association of *GSTT1* null variant (OR, 3.28; 95% CI, 1.31-8.26) with adult ALL but no association of *GSTM1* null genotype. The participants of this study were Caucasians.^21^ Results from another study involving 36 adult ALL patients from Turkey were similar for *GSTM1* null genotype but were contradictory for *GSTT1* as they showed negative association of *GSTT1* deletion with ALL (OR, 0.2; 95% CI, 0.05-0.9).^22^ However, the sample size of these studies was not large enough to define the frequency of these alleles among ALL patients in population-specific manner. A study with a larger sample size, 192 ALL patients, from India revealed no association of either *GSTM1* or *GSTT1* null genotype with ALL. The results of this study are comparable to our findings, however, the patients included in the study were all under 25 years of age.^23^ A meta-analysis comprising 23 studies from different Asian countries illustrated significant association of *GST* null polymorphisms with acute leukemia risk in children while no association with adults.^24^ This difference in results depict geographic variations in *GST* null allele distribution and its association with ALL.

## CONCLUSION

In conclusion, results from the current study suggest no statistically significant association of *GSTM1* null, *GSTT1* null, and *GSTM1/GSTT1* double null genotypes with ALL in adult patients from Pakistan. ALL risk may not only be affected by genetic factors but also with environmental factors. Therefore, studies with a better study design and a larger sample size including environmental exposure data in addition to genetic variants of xenobiotic metabolism are required to provide more insight into the etiology of adult ALL.
